# Addressing anaemia in adivasi women of reproductive age: urgent policy imperatives for Karnataka

**DOI:** 10.3389/fgwh.2025.1684392

**Published:** 2025-10-24

**Authors:** Prafulla Shriyan, Rahul Amruthapuri, Chandrashekar Kottagi, Prashanth N. Srinivas, Giridhara R. Babu, Tanya Seshadri, Pooja Aggarwal, Deepa Bhat, Suresh S. Shapeti

**Affiliations:** 1 Indian Institute of Public Health, Public Health Foundation of India, Bengaluru, India; 2 Centre of Adivasi Health, Institute of Public Health, Bengaluru, India; 3 Department of Population Medicine, College of Medicine, QU Health, Qatar University, Doha, Qatar; 4 Department of Anatomy, JSS Medical College, Mysuru, India

**Keywords:** anaemia, nutrition, cohort, Adivasi women, hemoglobinopathy, thalassemia trait

## Abstract

The policy brief analyses program implementation gaps among Adivasi population in Southern Karnataka to support India's ‘Anemia Mukt Bharat’ and Karnataka's ‘Anemia Mukt Poushtika Karnataka’ initiatives which aims to improve anemia and nutrition status of the population This analysis is based on a Adivasi birth cohort. Preliminary results show higher underweight and anemia prevalence in Adivasi women than National Family Health Survey-5 estimates. A significant proportion of women also have thalassemia traits, suggesting a genetic predisposition. Given the burden of sickle cell disease and the undernutrition, an intersectoral approach is needed. A multistakeholder committee, including Adivasi women, experts from public health and nutrition, others are crucial to design culturally appropriate initiatives and prioritize hemoglobinopathy screening, ensuring effective strategies for this vulnerable population.

## 1 Introduction

Anemia has been a persistent health issue in India, affecting a significant portion of the population throughout their life course (1). It manifests in many forms, including iron deficiency anemia (IDA), deficiency of Vitamin B12 or folate, dimorphic anemia (caused by multiple deficiencies) and hemolytic anemia [linked to genetic disorders like sickle cell disease (SCD) and thalassemia]. Iron deficiency anemia among women of reproductive age group is interlinked with functional impairment affecting oxygen transport, infection and inflammation (2), thermoregulation (3), immune function, and cognitive function (4), maternal and birth outcomes (5–7). Preventing iron deficiency in women of childbearing age is essential to prevent poor child health outcomes such as low birth weight, preterm birth, delayed cognitive and motor development, and poor academics in childhood and adolescence in later life (8–12).

In India, with the launch of the Anaemia Mukt Bharat (AMB), a national initiative in 2018, India has committed to tackling this pressing public health concern by reducing anemia among women of reproductive age group (13) The Government of Karnataka through AMB, has committed to Anaemia Mukth Poushtika—Karnataka (AMP-K) (Anemia-free Nutritious Karnataka) and is following the national program's guidelines. AMP-K aims to improve women's and children's health and nutrition through increased community awareness, screening, treatment, and monitoring (13, 14).

Although India has made strides to combat anaemia, the prevalence remains high, reflecting persistent inequities in many regions (15). The National Family Health Survey round five(2019–2020) reveals an increase in anaemia prevalence in Karnataka. Anaemia among women aged 15–49 increased from 44.8% to 47.8% despite of interventions like iron-folic acid supplementation under Anaemia Mukt Bharat.

Tribal populations who represent 7% of Karnataka's population are more vulnerable to anemia due to additional challenges such as social disadvantage, structural and historical issues (16, 17), residing in remote areas with poor healthcare access (18), sanitation (19) and higher burden of sickle cell disease (18%–20%) trait prevalence and 1%–2% disease prevalence (20) and being underweight (21).

From a public policy and practice standpoint to address the high prevalence of anemia, it is important to assess the situation of the AMB initiatives that are being implemented at the community level for the tribal populations in Karnataka to identify effective strategies for screening for anemia and management. This article provides a overview of iron deficiency anemia in women of reproductive age group from tribal populations in one district of Karnataka with the goal of informing interested stakeholders, including policymakers about the factors to be considered for the reduction of iron deficiency anemia among tribal populations.

Chiguru is a Adivasi cohort study established in Chamarajanagar district, in partnership between the Institute of Public Health, Bengaluru, JSS Medical College Mysore and the Indian Institute of Public Health, Public Health Foundation of India, Bengaluru. Chiguru is a Kannada word referring to ‘early leaves from a new plant’. The name symbolizes new beginnings and life, reflecting the focus of our cohort. Drawing on early findings from the Chiguru Adivasi cohort, we offer evidence-informed recommendations to strengthen anemia prevention and control among Adivasi populations.

## 2 Chiguru adivasi birth cohort: key observations

The word “Adivasi” is used as label for tribal communities for forest associated tribal communities who prefer this label to indicate their ownership over areas they inhabit today. The Chiguru Adivasi birth cohort was established in Chamarajanagar district's Adivasi villages in May 2023. As of 2025, the cohort has enrolled 2,730 Adivasi individuals (≤70 years) to investigate the effect of parental use of substance use on child growth and developmental outcomes, including 1,406 women, of which 1,136 (80%) are of reproductive age (18–49 years), including postnatal women 821 (72%) and other family members 315 (28%). Here the substance use is defined as use of substances like smoking, smokeless tobacco and alcohol among the parents of the newborn child enrolled in the cohort study. While the standard definition of reproductive age is 15–49 years, we included women aged 18–49 years in the study to ensure ethical compliance. This decision was made in view of obtaining voluntary written consent from the individuals in this difficult geography. The cohort captures the anthropometric profile of the Adivasi families by assessing the weight, height, and blood hemoglobin status of the household members of the enrolled Adivasi women along with other data such as substance use. So far, we have completed anthropometric assessment in 60 percent (*n* = 681) of the enrolled women in the reproductive age group.

**Figure 1:**
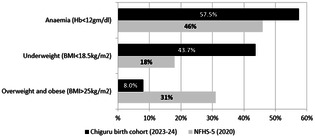
Comparison of nutritional status of non-pregnant adivasi women(chiguru cohort) with NFHS data in chamarajanagar district.

In this brief, we have compared the nutritional indicators of Adivasi women of reproductive age group from our study against the National Family Health Survey—round five district level fact sheet for Chamarajanagar. It is important to note that the NFHS-5 data is inclusive of both tribal and non-tribal populations, while our policy brief focuses specifically on the Adivasi tribals residing in Chamarajanagar district. The purpose of this brief is to characterise the nature of social disadvantages faced by Adivasi tribal populations. Given that forest associated tribal communities’ population is small, we anticipate that district level averages may not adequately reflect the nutritional status appropriately. Anthropometric profiles from the Adivasi cohort revealed that 46 percent of women in the reproductive age group are underweight(BMI < 18.5 kg/m^2^). This is in contrast to the National Family Health Survey-5 for Chamarajanagar district, which showed a declining trend in underweight women from 26 percent in 2015 to 18 percent in 2019–2020 (Figure 1) (22).

Among those who consented to provide venous blood (396/1,136), 60% of reproductive-age women were anaemic (haemoglobin <12 g/dl), surpassing the 46% district estimate (Hb cut-off: 12 gm/dl) as shown in Figure 1. We also performed nutritional biomarker assessments among postnatal women 90 days after delivery (*n* = 297). Specifically, we estimated ferritin and the amount of soluble Transferrin Receptor(sTfR) protein that transports iron from blood to tissue cells, in an accredited laboratory in Bengaluru. Test reports showed that 20 percent of the postnatal women had insufficient iron storage (ferritin <13 ng/ml). Further, 64 percent of them exhibited elevated levels of soluble transferrin receptor protein (sTfR > 4.13 mg/L), which represents the true demand for iron requirement in the body (23, 24). This suggests underestimation of iron insufficiency if relying on haemoglobin levels alone without assessing other biomarkers. Adding to the complexity, based on Mentzer Index estimates, one-third of tested women carry the thalassaemia trait, underscoring the importance of routine screening for haemoglobinopathies (25).

The difference in nutritional and anemia status of women between the national survey and the cohort data can be because of multiple reasons not limited to the cohort data being representative of the Adivasi women residing in Chamarajanagar District. Although National Family Health Survey round five report provides valuable insights it is important to note that it does not sufficiently represent the situation of Adivasi population.

We observed that many Adivasi villages are located in remote forest peripheries, limiting access to healthcare services and timely anaemia treatment due to barriers to road access by forest regulations and poor implementation of Forest Rights Act (26)^1^. We also noticed unique cultural beliefs and dietary preferences, that can influence anaemia risk (27). Approximately 29% of adults use tobacco in some form, and 20% consume alcohol, which can exacerbate micronutrient deficiencies and impede iron absorption (28–32).

## 3 Policy options and implications

Adivasi women in Karnataka continue to experience a high burden of anemia, despite six years of the AMB program. This might be due to systemic, socioeconomic, and implementation challenges that have limited AMB's success. The program's top-down, one-size-fits-all approach does not account for the unique dietary habits (limited access to animal protein), traditional health practices, and prevalent hemoglobinopathies within these communities. Moreover, calorie- and protein-rich food distribution through the public distribution system is poorly integrated with AMB initiatives. The National Sickle Cell Anemia Elimination Mission (NSCAEM), which addresses sickle cell disease among Adivasi populations, is also hampered by low screening rates, lack of awareness, and reluctance toward medical management. Greater integration between these programs is essential to reduce the disproportionate burden of anemia in this vulnerable group.

Unaddressed burden of iron deficiency anemia among women of reproductive age group will increase the risk of maternal morbidity and poor child health outcomes. Thus, iron deficiency anemia among women of reproductive age group affects not only women but also their children, families and society as well.

## 4 Policy and practice recommendations

Considering the emerging evidence from the context described above, there is an urgent necessity for policymakers to consider and act upon.

### 4.1 Implementing a multistakeholder and multimodal intervention

The evidence from blood investigations reveals a need for iron supplementation. However, considering the prevalence of sickle cell disease trait in this population, there is a need for sickle cell screening prior to starting iron supplements.

To address this complexity, we recommend a multimodal and intersectoral approach that can address the underlying factors contributing to anemia. Specifically, we recommend an multistakeholder committee at the State level with flexibility at the district level comprising of the government, academia, civil society, and community-based organizations including Adivasi women. This committee can include various government departments along with experts in public health, nutrition, and Adivasi health. The mandate of this committee should be to develop a culturally tailored policy roadmap to compliment ongoing AMP-K activities by incorporating local cultural and dietary practices, existing public food distribution channels and integrative strategies (e.g., Iron supplements, deworming and food fortification) while prioritizing screening for sickle cell disease.

### 4.2 Modifying the AMP-K program for individuals and populations which have with hemoglobinopathies

Incorporating routine screening for sickle cell disease is important in Adivasi communities. Staff in health centres implementing Anemia Mukt Poushtika Karnataka need to be sensitized about the sicke cell disease, screening methods, and treatment options for women who have anemia due to sickle cell disease. This initiative should find synergies with the National Sickle Cell Anaemia Elimination program and offer holistic screening for anaemia, counselling and follow-up support to families identified at risk. This will not only address the challenges in the study area but also offer model for screening in other vulnerable communities.

### 4.3 Contextual adaptation to the geographical and cultural context settings of adivasi people

In districts such as Chamarajanagar, a majority of the Adivasi community lives in forest periphery or in reserved forest areas associated with remoteness and geographic isolation. This may hinder their access to healthcare services (see text footnote 1) which is essential for treating anemia. As a result, aligning programmatic strategies with the geographical realities of forest peripheries is essential. Promoting community-based outreach clinics, mobile health units, and task shifting approaches can help to ensure timely screening, diagnosis and treatment.

Adivasi communities have unique cultural practices, beliefs, and dietary habits. Targeted information education and communication outreach initiatives including role plays and street play to convey information emphasizing early screening, iron supplementation and dietary modification can be attempted. By customizing health education messages to suit the cultural context and language preferences of Adivasi populations, we can ensure that health information is accessible and understandable. Collaborating with local community leaders can facilitate health literacy and reinforce positive health seeking behaviour.

### 4.4 School based nutrition education

Along with the current existing nutritional interventions provided through mid-day meal program and weekly iron and folic acid supplementation program, we recommended a continuous, school based educational program to raise awareness on anemia, causes and symptoms and the importance of iron rich food using short animated videos. This will empower both individuals and community with crucial knowledge for anemia prevention.

#### 4.4.1 Addressing other factors associated with anemia

To address undernutrition and anemia that often accompanies substance use AMP-K needs to integrate screening for women for substance use and connect them to brief interventions like health education from nurses (33), self help programs (34), and strengthening of community drug treatment centers (35) at government health facilities. Staff at the health care facility can be trained to discuss tobacco and alcohol cessation for those who need such services and referring them to respective community drug treatment centers/clinics. Furthermore, fostering community support groups, leveraging local networks to raise awareness about the impact of substance use on iron absorption and overall nutritional status can also help (36).

The risk of anemia among women who are underweight is more when compared to those with normal weight, as indicated byevidence from several low- and middle-income countries including India (37–39). Addressing this issue requires intersectoral interventions that extend beyond healthcare. Such efforts include improving access to clean water and sanitation, tackling open defecation and supporting income generating activities among women of reproductive age group. So that this will make them empower to make healthier choices recognising that their well-being is linked to the broader socio economic and environmental factors.

## 5 Conclusion

Evidence from the Chiguru Adivasi birth cohort points to significantly higher rates of underweight status and anaemia among Adivasi women compared to district-level National Family Health Survey round five estimates. Current “one-size-fits-all” policies and policy actions may not effectively address the distinctive sociocultural, economic, and geographical barriers faced by Adivasi communities.

We therefore urge policymakers and stakeholders to prioritize tailored multimodal and multisectoral interventions, guided multistakeholder committee, that integrate culturally sensitive approaches, address underweight, substance use, and incorporate routine screening for sickle cell disease. Successful implementation in Chamarajanagar can serve as a blueprint for extending similar targeted programs statewide, thereby accelerating progress toward a truly Anaemia Mukt Poushtika Karnataka and ultimately improving the health and well-being of vulnerable Adivasi populations.

## Footnotes

1 Anika Juneja PNS, Garimella S, Hurtig A-K. Forest neighbourhoods and healthcare access for adivasi communities in India: a critical interpretive synthesis. *J Community Syst Health*. (2025).

## References

[1] Preethi V Hemalatha V Arlappa N Thomas MB Jaleel A. Trends and predictors of severe and moderate anaemia among children 2 aged 6–59 months in India: an analysis of three rounds of national family health survey 3 (NFHS) data. BMC Public Health. (2024) 24(1):2824. 10.1186/s12889-024-20328-9 39402527 PMC11476725

[2] Nemeth E Rivera S Gabayan V Keller C Taudorf S Pedersen BK IL-6 mediates hypoferremia of inflammation by inducing the synthesis of the iron regulatory hormone hepcidin. J Clin Invest. (2004) 113(9):1271–6. 10.1172/JCI20945 15124018 PMC398432

[3] Clark SF. Iron deficiency Anemia. Nutr Clin Pract. (2008) 23(2):128–41. 10.1177/0884533608314536 18390780

[4] Silva B Faustino P. An overview of molecular basis of iron metabolism regulation and the associated pathologies. Biochim Biophys Acta. (2015) 1852(7):1347–59. 10.1016/j.bbadis.2015.03.011 25843914

[5] Anaemias W On N, W.H. Organization. Nutritional Anaemias: Report of a WHO Scientific Group [Meeting Held in Geneva from 13 to 17 March 1967]. Geneva, Switzerland: World Health Organization (1968).

[6] Sazawal S Dhingra U Dhingra P Dutta A Shabir H Menon VP Efficiency of red cell distribution width in identification of children aged 1–3 years with iron deficiency anemia against traditional hematological markers. BMC Pediatr. (2014) 14:8. 10.1186/1471-2431-14-8 24428927 PMC3897999

[7] Finkelstein JL Kurpad AV Thomas T Srinivasan K Duggan C. Vitamin B12 status in pregnant women and their infants in south India. Eur J Clin Nutr. (2017) 71(9):1046–53. 10.1038/ejcn.2017.29 28402324 PMC8141370

[8] Gambling L Danzeisen R Fosset C Andersen HS Dunford S Srai SK MCArdle HJ. Iron and copper interactions in development and the effect on pregnancy outcome. J Nutr. 2003 133(5 Suppl 1):1554S–6S. 10.1093/jn/133.5.1554S 12730464

[9] Allen LH. Anemia and iron deficiency: effects on pregnancy outcome. Am J Clin Nutr. (2000) 71(5 Suppl):1280s–4. 10.1093/ajcn/71.5.1280s 10799402

[10] Hansen M Singh G Barzi F Brunette R Howarth T Morris P Maternal anaemia in pregnancy: a significantly greater risk factor for anaemia in Australian aboriginal children than low birth weight or prematurity. Matern Child Health J. (2020) 24(8):979–85. 10.1007/s10995-020-02913-7 32495246

[11] Beard J. Iron deficiency alters brain development and functioning. J Nutr. (2003) 133(5):1468S–72. 10.1093/jn/133.5.1468S 12730445

[12] Milman N. Postpartum anemia II: prevention and treatment. Ann Hematol. (2012) 91:143–54. 10.1007/s00277-011-1381-2 22160256

[13] Bharat AB. Available online at: https://anemiamuktbharat.info/ (Accessed March 27, 2024)

[14] Ranganath TS Ashutosh Vaidya S. Anemia free Karnataka—a way forward. RGUHS Nat J Public Health. (2023) 8:6–7. 10.26463/rnjph.8_4_1

[15] Subramanian SV Joe W Kumar A Rana MJ Tripathi N Arshad R Anemia mukt bharat, 35 in insights from ranking of key performance indicators, NFHS 2015–16 and NFHS 2019–21. 36 2022, Demographic and Health Surveys and International Institute for Population Sciences 37 for the Data: India

[16] Xaxa V. Report on the High Level Committee on Socio-Economic, Health and Educational Status of Tribal Communities of India. New Delhi: Ministry of Tribal Affairs, Government of India (2014).

[17] Thresia CU Srinivas PN Mohindra KS Jagadeesan CK. The health of indigenous populations in south Asia: a critical review in a critical time. Int J Health Serv. (2022) 52(1):61–72. 10.1177/0020731420946588 32787539 PMC7611999

[18] Khambalia AZ Aimone AM Zlotkin SH. Burden of anemia among indigenous populations. Nutr Rev. (2011) 69(12):693–719. 10.1111/j.1753-4887.2011.00437.x 22133195

[19] Larsen DA Grisham T Slawsky E Narine L. An individual-level meta-analysis assessing the impact of community-level sanitation access on child stunting, anemia, and diarrhea: evidence from DHS and MICS surveys. PLoS Negl Trop Dis. (2017) 11(6):e0005591. 10.1371/journal.pntd.0005591 28594828 PMC5464528

[20] Urade B. Sickle cell gene (HbS) scenario in tribal India. J Health Med Inform. (2012) 3(3):1–6. 10.4172/2157-7420.1000114

[21] Pujari S Thunga G Vijayanarayana K Shetty RS Mundkur SC Devi ES Exploring the health dynamics: a comprehensive assessment of malnutrition 1 among tribal children through anthropometric and laboratory evaluations in southern 2 Karnataka. Clin Epidemiol Glob Health. (2025) 35:102159. 10.1016/j.cegh.2025.102159

[22] The International Food Policy Research Institute (IFPRI). District Nutrition Profile. Bengaluru, Karnataka: IFPRI (2022).

[23] Beard J. Indicators of the Iron status of Populations: Free Erythrocyte Protoporphyrin and Zinc Protoporphyrin; Serum and Plasma Iron, Total Iron Binding Capacity and Transferrin Saturation; and Serum Transferrin Receptor. Oxford: Oxford University Press (2008).

[24] Baynes RD Shih YJ Hudson BG Cook JD. Production of the serum form of the transferrin receptor by a cell membrane-associated serine protease. Proc Soc Exp Biol Med. (1993) 204(1):65–9. 10.3181/00379727-204-43635 8372098

[25] Tabassum S Khakwani M Fayyaz A Taj N. Role of mentzer index for differentiating iron deficiency anemia and beta thalassemia trait in pregnant women. Pak J Med Sci. (2022) 38(4Part-II):878–82. 10.12669/pjms.38.4.4635 35634613 PMC9121960

[26] Anika Juneja PNS Garimella S Hurtig A-K. Forest neighbourhoods and healthcare access for adivasi communities in India: a critical interpretive synthesis. J Community Syst Health. (2025). 10.36368/jcsh.v2i2.1187 PMC7619491 42732440

[27] Rai ND Benjaminsen TA Krishnan S Madegowda C. Political ecology of tiger conservation in India: adverse effects of banning customary practices in a protected area. Singap J Trop Geogr. (2019) 40:124–39. 10.1111/sjtg.12259

[28] Ome-Kaius M Unger HW Singirok D Wangnapi RA Hanieh S Umbers AJ Determining effects of areca (betel) nut chewing in a prospective cohort of pregnant women in Madang province, Papua New Guinea. BMC Pregnancy Childbirth. (2015) 15:177. 10.1186/s12884-015-0615-z 26286026 PMC4543471

[29] Kader M. Association Between Betel nut Consumption and Folate Deficiency among Pregnant Women in Rural Bangladesh. Mumbai: Medknow Publications (2013).

[30] Cimolai N Cimolai T. Severe iron deficiency anemia and gastrointestinal dysfunction associated with ingestion of pan masala. J Diet Suppl. (2008) 5(3):305–9. 10.1080/19390210802414352 22432465

[31] Lieb M Palm U Hock B Schwarz M Domke I Soyka M. Effects of alcohol consumption on iron metabolism. Am J Drug Alcohol Abuse. (2011) 37(1):68–73. 10.3109/00952990.2010.535584 21091174

[32] Lindenbaum J Roman M. Nutritional anemia in alcoholism. Am J Clin Nutr. (1980) 33(12):2727–35. 10.1093/ajcn/33.12.2727 7001890

[33] Kamal K Sunita S Karobi D Abhishek G. Nurse-delivered screening and brief intervention among college students with hazardous alcohol use: a double-blind randomized clinical trial from India. Alcohol Alcohol. (2020) 55(3):284–90. 10.1093/alcalc/agaa014 32103254

[34] Schaub MP Tiburcio M Martínez-Vélez N Ambekar A Bhad R Wenger A The effectiveness of a web-based self-help program to reduce alcohol use among adults with drinking patterns considered harmful, hazardous, or suggestive of dependence in four low- and middle-income countries: randomized controlled trial. J Med Internet Res. (2021) 23(8):e21686. 10.2196/21686 34448710 PMC8433861

[35] Dhawan A Rao R Ambekar A Pusp A Ray R. Treatment of substance use disorders through the government health facilities: developments in the “drug de-addiction programme” of ministry of health and family welfare, government of India. Indian J Psychiatry. (2017) 59(3):380–4. 10.4103/psychiatry.IndianJPsychiatry_19_17 29085101 PMC5659092

[36] Saxena S Singh PK Singh L Kashyap S Singh S. Smokeless tobacco use and public health nutrition: a global systematic review. Public Health Nutr. (2023) 26(1):46–55. 10.1017/S1368980022001331 35618706 PMC11077452

[37] Chakrabarty M Singh A Singh S Chowdhury S. Is the burden of anaemia among Indian adolescent women increasing? Evidence from Indian demographic and health surveys (2015–21). PLOS Glob Public Health. (2023) 3(9):e0002117. 10.1371/journal.pgph.0002117 37672528 PMC10482272

[38] Khan ZA Khan T Bhardwaj A Aziz SJ Sharma S. Underweight as a risk factor for nutritional anaemia–a cross-sectional study among undergraduate students of a medical College of Haryana. Indian J Community Health. (2018) 30(1):63–9. 10.47203/IJCH.2018.v30i01.011

[39] Lakew Y Biadgilign S Haile D. Anaemia prevalence and associated factors among lactating mothers in Ethiopia: evidence from the 2005 and 2011 demographic and health surveys. BMJ open. (2015) 5(4):e006001. 10.1136/bmjopen-2014-006001 25872935 PMC4401847

